# Age-specific characterization of spinal cord injuries over a 19-year period at a Japanese rehabilitation center

**DOI:** 10.1371/journal.pone.0195120

**Published:** 2018-03-29

**Authors:** Mitsunori Toda, Eiji Nakatani, Kaoru Omae, Masanori Fukushima, Takaaki Chin

**Affiliations:** 1 Department of Physical Medicine and Rehabilitation, Hyogo Rehabilitation Center, Akebono-cho, Nishi-ku, Kobe, Japan; 2 Translational Research Informatics Center, Foundation for Biomedical Research and Innovation, Manatojima-minamimachi, Chuo-ku, Kobe, Japan; 3 Department of Biostatistics and Data Science, Osaka University Graduate School of Medicine, Yamadaoka, Suita, Osaka, Japan; 4 Department of Rehabilitation Science, Kobe University Graduate School of Medicine in Hyogo Rehabilitation Center, Kobe, Japan; University of Illinois at Urbana-Champaign, UNITED STATES

## Abstract

Regional demographics of spinal cord injuries (SCIs) are fundamental to identifying and implementing appropriate preventive measures. The current study was conducted as a longitudinal analysis of all patients with SCIs admitted to the Hyogo Rehabilitation Center over a 19-year period. The sex and age of the patient, time and nature of injury (i.e., cause, level, and extent), and period from injury to admission were evaluated retrospectively. Pertinent tests, including Poisson regression analysis, and the Cochran–Armitage, Kruskal–Wallis, and chi-square tests, were applied to assess demographic variables, with statistical significance set at *p* < 0.05. Between 1995 and 2013, a total of 632 patients with SCIs (predominantly male and largely < 60 years old) were admitted to our center for rehabilitation. Although the male: female ratio remained unchanged throughout the study period, the ratio of older adults increased over time. In assessing the cause of injury, the majority of the patients involved in road traffic accidents were aged ≤ 44 years, whereas patients aged ≥ 45 years accounted for the majority of low-distance falls and disease-related SCIs, the proportions of which gradually increased. Complete paralysis and paraplegia primarily occurred in patients aged ≤ 44 years, whereas the majority of incomplete injuries and tetraplegia were limited to those aged ≥ 45 years. The patient age at the time of SCI and the nature of the injury sustained were interrelated. Age-specific strategies thus offered the best means of preventing/reducing the incidence of SCIs in Hyogo prefecture.

## Introduction

Spinal cord injury (SCIs) is an inherently serious condition that affects the expectancy and quality of life and exacts a heavy economic toll. Since there are currently no adequate restorative therapies, prevention remains the best approach. Thus, epidemiological studies of SCIs are essential to provide a basis for defining and implementing appropriate preventive measures [1].

During 1990–1992, the Japan Medical Society of Spinal Cord Lesions conducted a nationwide epidemiological survey of acute traumatic SCIs. Based on their postal questionnaire, the annual incidence of SCI was reportedly 40.2/1,000,000 in the population [2]. Most cases (75%) involved cervical injury, with patients in their seventh decade considered most vulnerable, followed by those in their third decade [2,3]. Recently, Katoh et al. [4] also documented the incidence of traumatic SCIs in a rural population in Japan during 2011 and 2012, and found that the incidence of incomplete cervical SCIs was greater than that previously reported. A nationwide study in the United States suggested that the incidence of SCI increased with increasing age, presumably due to an increase in the frequency of falls [5]. However, to the best of our knowledge, there have been no published studies of an increase in SCIs associated with the aging population in Japan that exceeded a 10-year period.

With a total population of approximately 127 million, Japan now has a burgeoning number of older adults (≥ 65 years), which increased from 14.6% in 1995 to 25.0% in 2013 [6]. Hyogo is the seventh largest prefecture in Japan, with a total population of approximately 5.56 million in 2013. The Hyogo Rehabilitation Center, located in the southeast Hyogo prefecture, is one of the key referral hospitals for rehabilitation after SCI. Local patients with relatively severe dysfunction, whether traumatic or non-traumatic in etiology, were likely to be referred to the Hyogo Rehabilitation Center after acute-phase SCI treatment.

This study entailed a longitudinal analysis of patients with SCIs admitted to the Hyogo Rehabilitation Center over a 19-year period (1995–2013). Patient characteristics (i.e., sex and age) and the nature of injuries (i.e., cause, level, and extent) were examined. A better understanding of groups at risk of SCI is needed to improve preventative strategies and management/healthcare services for patients with SCIs.

## Patients and methods

### Study population

All patients admitted to the Hyogo Rehabilitation Center between January 1, 1995, and December 31, 2013, for management of SCIs were eligible for study. All patients showed rehabilitative potential. Within its catchment area, the center is one of the core public rehabilitation centers for patients with SCIs.

### Data collection

Records of each study participant were reviewed by one of the authors (MT) in collaboration with a physical therapist. The data collected for analysis included the following: patient parameters (i.e., sex and age), time of injury, nature of injury (i.e., cause, level, and extent), and period from injury to admission to the Hyogo Rehabilitation Center. Extent of injury was further categorized as complete or incomplete, according to American Spinal Injury Association (ASIA) classification. The time of injury was also confined to four intervals (1995–1998, 1999–2003, 2004–2008, and 2009–2013), and causes of injury were stratified as follows: road traffic accidents (RTAs), falls, diseases, sports, impact due to heavy/falling objects, suicide attempts, and other injuries. Falls were then categorized by height, as high (> 1 m) or low (≤ 1 m), depending on the distance from ground level. SCIs due to non-traumatic causes were classified by related diseases, such as vascular disorders (i.e., hemorrhage, vascular malformation, and ischemia), degenerative ailments of the vertebral column (i.e., spondylosis, spinal stenosis, and ossification of posterior longitudinal ligament [OPLL]), tumors (benign or malignant), bacterial or viral infection, and inflammatory and autoimmune diseases. The period from injury to admission to the center was categorized by duration as follows: < 4 months, 4–7 months, or ≥ 8 months. Neurological status was assessed using the ASIA standard [7] as follows: motor scores, level of neurological deficit (i.e., cervical, tetraplegia; thoracic or lumbar paraplegia), and the ASIA impairment scale (AIS). The level and completeness of SCI were determined on admission to the Hyogo Rehabilitation Center, usually within weeks to months after the injury. Patients with incomplete lesions retained sacral-segment motor and sensory function (sacral sparing: ASIA B, C, D). Data classifications conformed to the International Spinal Cord Injury Core Data Set [7] and International Spinal Cord Injury Core Data Sets for Non-traumatic Spinal Cord Injury [8].

### Statistical analysis

Continuous and categorical variables were expressed as mean ± standard deviation (SD) and frequency or percentage, respectively. A binary test with a null hypothesis (i.e., *p* = 0.5) was applied to pertinent variables, such as sex. The four study period subsets (i.e., 1995–1998, 1999–2003, 2004–2008, and 2009–2013) were referred to as period classes. Poisson regression analysis was used to compare numbers of patient admissions by period class. The Cochran–Armitage trend test was used to investigate rate shifts during the periods specified. Finally, the Kruskal–Wallis (Wilcoxon rank sum test for two groups) and chi-squared tests were applied for between-group comparisons for continuous and categorical variables, respectively. Statistical significance was set at *p* < 0.05. All computations were performed using standard software, including SAS v9.3 (SAS Institute Inc., Cary, NC, USA) and R freeware v3.3.2 (www.r-project.org).

### Ethics

This study was conducted in accordance with the Declaration of Helsinki. All patient data were unlinked and anonymous, ensured by the President of the Hyogo Rehabilitation Center. The study protocol and other relevant documents were reviewed and approved by the Institutional Review Board of the Hyogo Rehabilitation Center (No. 1524, 25/Feb/2016) and Ethics Committee of the Foundation for Biomedical Research (No. 15-10-12, 15/Jun/2016).

## Results

### Patient stratification and demographics

Following traumatic or non-traumatic SCIs (NTSCIs), a total of 632 patients undergoing rehabilitation during the study period (1995–2013) were identified and included in this investigation (S1 Table). None of the patients admitted to the Hyogo Rehabilitation Center were excluded. The four established intervals for patient admissions were as follows: 1995–1998, 100 (15.8%); 1999–2003, 253 (40.0%); 2004–2008, 171 (27.1%); and 2009–2013, 108 (17.1%). The Poisson regression analysis (S2 Table) indicated that these rates differed significantly (*p* < 0.001).

S2 Table outlines the patient characteristics. Men and women accounted for 82.9% (524/632) and 17.1% (108/632) of the cohort, respectively, yielding a male: female ratio of 4.8:1.0 (*p* < 0.001). This ratio remained essentially unchanged throughout the entire study period (*p* = 0.248, S2 Table).

**Fig 1:**
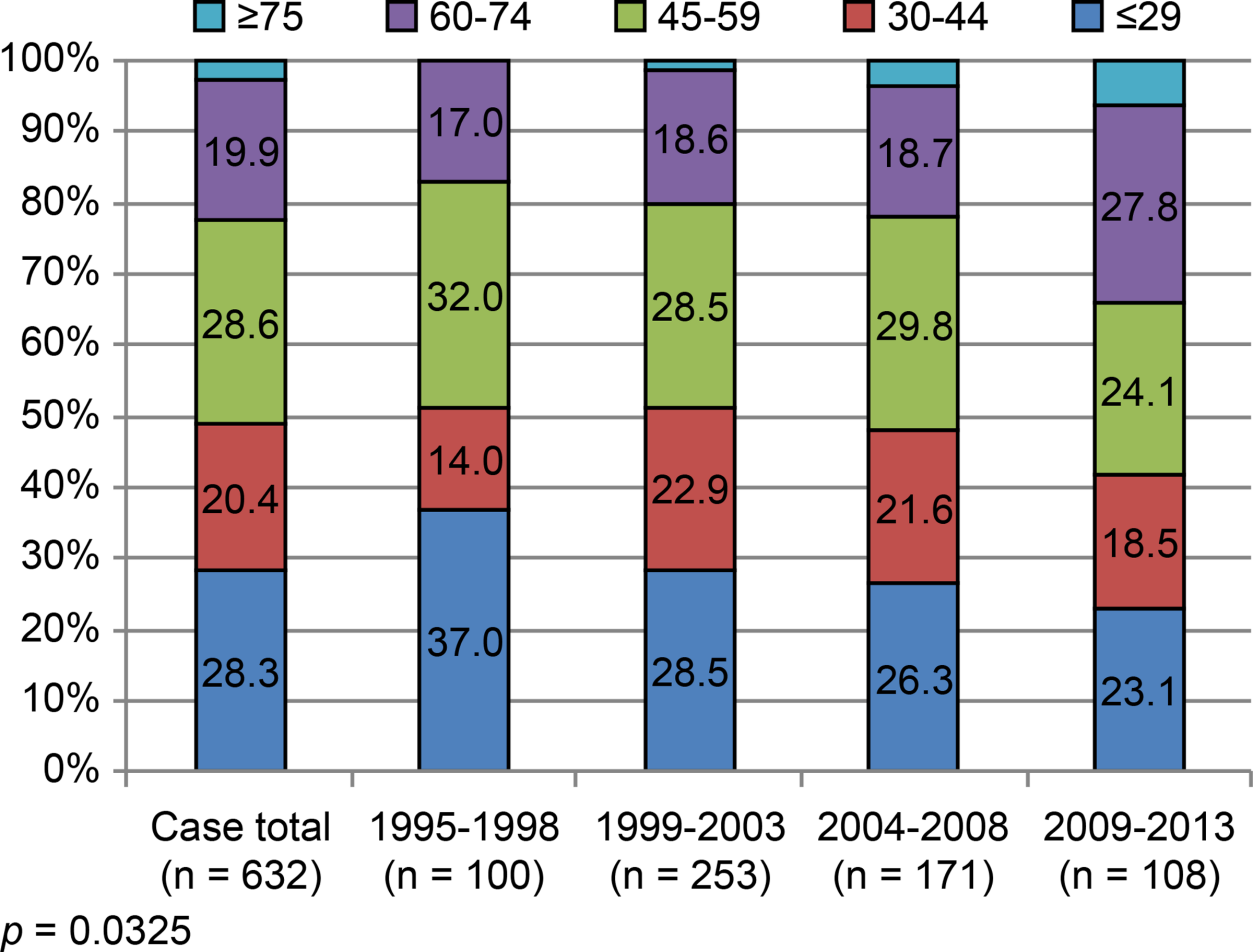
Age (years) at injury (distributions at set intervals).

In this patient population, age distribution showed a weak biphasic fluctuation. Patient subsets, defined by age, i.e., ≤ 29 years (179/632, 28.3%) and 45–59 years (181/632, 28.6%), displayed small peaks. Patients ≥ 60 years old comprised 22.6% (143/632) of the total cohort, and the ratio of older adults increased from one period to the next. Relative to the 1995–1998 period, the proportion of patients ≥ 60 years old during 2009–2013 was significantly higher (34.3% vs 17.0%; *p* = 0.003; Fig 1).

**Fig 2:**
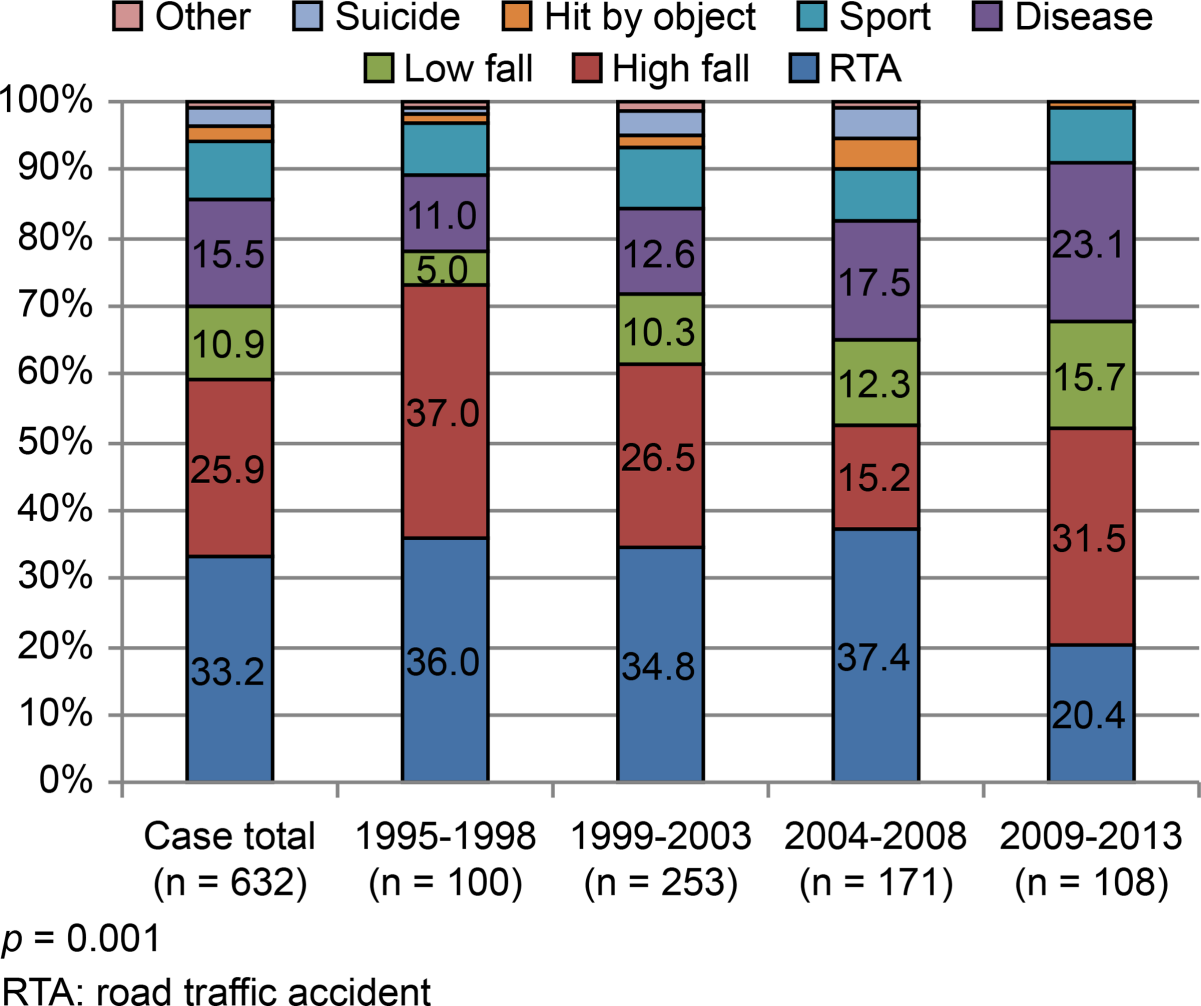
Cause of injury (distributions at set intervals).

RTAs constituted the most frequent cause of injury (33.2%, 210/632), followed by high-distance falls (25.9%, 164/632), various diseases (15.5%, 98/632), low-distance falls (10.9% 69/632), and sports (8.4%, 53/632) (Fig 2). During the 1995–2008 period, RTA was the most frequent cause of injury (accounting for approximately 36.0–37.4%), but the second most common cause (20.4%) during the 2009–2013 period; the rate of high-distance falls exceeded that of RTAs during the 2009–2013 period (Fig 2). High-impact traumas (i.e., RTAs and high-distance falls) were thus the leading causes of SCIs overall (59.1%, Fig 2). Throughout the study period, the frequency distributions according to the cause of injury differed significantly (*p* = 0.001, Fig 2). Although the proportion of SCIs attributable to these causes gradually declined (1995–1998: 73.0%; 2009–2013: 51.9%; *p* < 0.001), they were still the most common causes. Diseases ranked third as cause of SCIs (NTSCIs 15.5%), and their importance gradually increased over time (*p* = 0.005; S2 Table). Causes of NTSCIs included degenerative disorders, such as spinal spondylosis and OPLL (29.6%, 29/98), vascular diseases (28.6%, 28/98), tumors (24.5%, 24/98), bacterial/viral infections (15.3%, 15/98), and inflammatory/autoimmune conditions (1.0%, 1/98). In one instance, no underlying cause was evident. Although low-distance falls (on ground level) accounted for 10.9% (69/632) of SCIs (Fig 2), the proportion of patients with this cause gradually increased throughout the study period, from only 5.0% in 1995–1998 to 15.7% in 2009–2013 (*p* = 0.012, Fig 2).

Cervical (tetraplegic) and thoracic or lumbar (paraplegic) SCIs accounted for 56.5% (357/632), 29.0% (183/632), and 14.6% (92/632) of patients, respectively (S2 Table). Rates of cervical injuries tended to decline gradually over time (1995–1998: 63.0%; 2009–2013: 54.6%; *p* = 0.149) (S2 Table), whereas rates of lumbar injuries tended to increase gradually (1995–1998: 9.0%; 2009–2013: 18.5%; *p* = 0.117).

**Fig 3:**
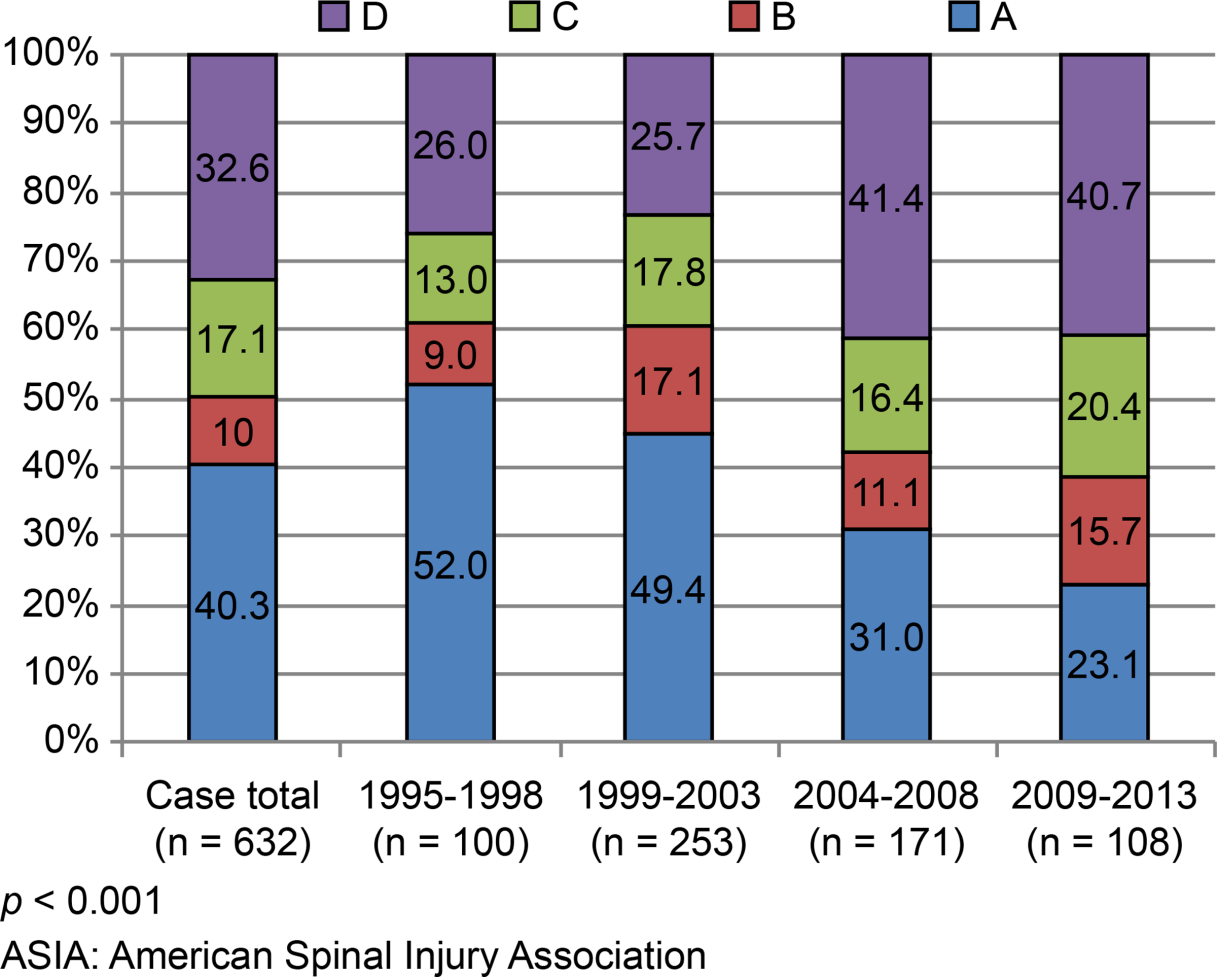
ASIA impairment scale (distributions at set intervals).

As for the extent of injury, complete (AIS-A) and incomplete (AIS-B, -C, and -D) paralysis accounted for 40.3% (255/632) and 59.7% (377/632) of patients, respectively (Fig 3). The frequency distributions of AIS scores at admission differed significantly during the study period (*p* < 0.001, Fig 3). The rate of AIS-A declined from 52.0% (1995–1998) to 22.2% (2009–2013), whereas that of AIS-B and AIS-C increased from 22.0% (1995–1998) to 35.7% (2009–2013). Similarly, the rate of AIS-D rose from 26.0% (1995–1998) to 40.7% (2009–2013) (Fig 3).

**Fig 4:**
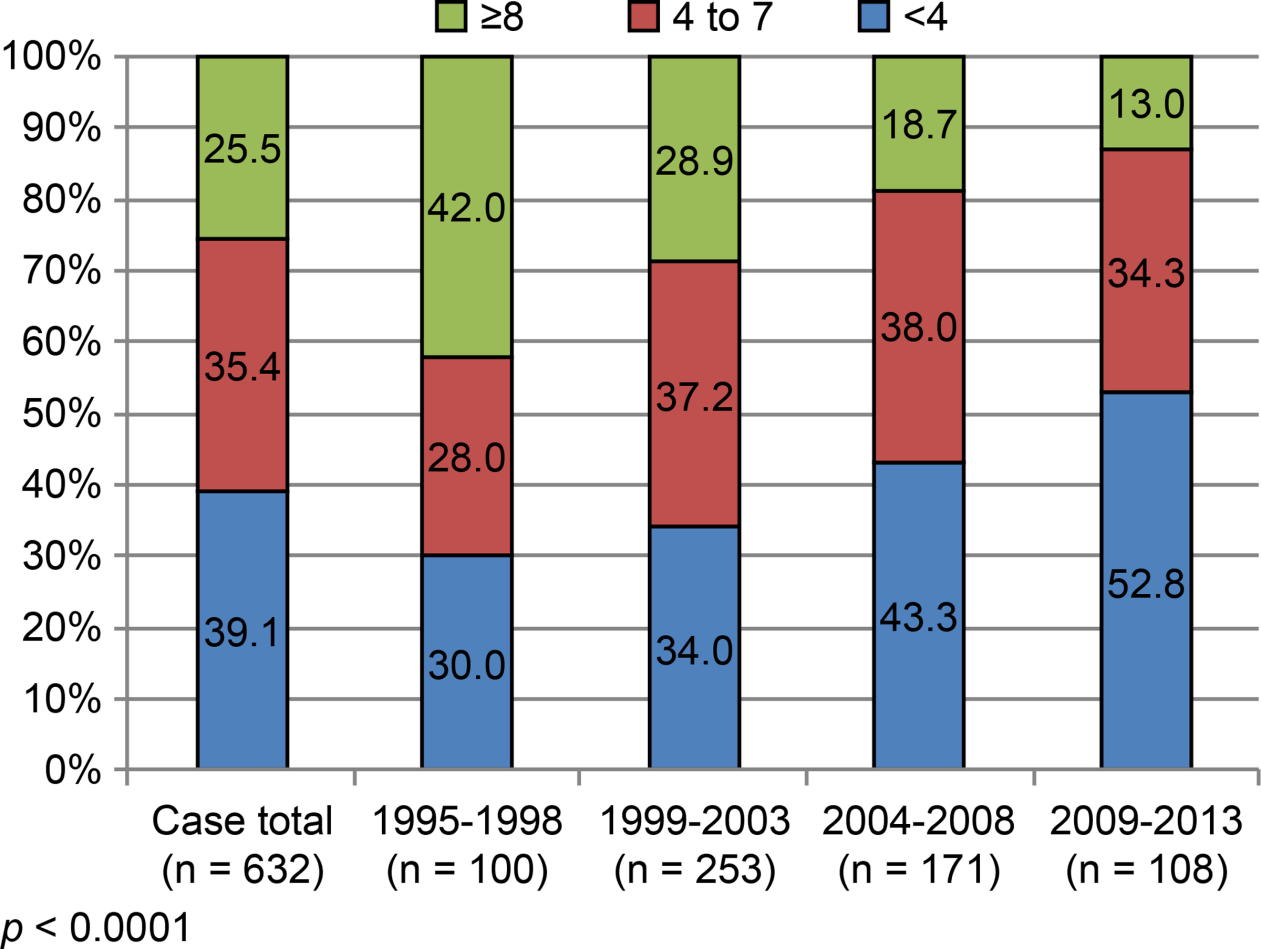
Time (month) from injury to admission (distributions at set intervals).

Admissions within 4 months, between 4–7 months, and ≥ 8 months after injury accounted for 39.1% (247/632), 35.4% (224/632), and 25.5% (161/632) of patients, respectively (Fig 4). The frequency distributions of period from injury to admission also differed significantly during the study period (*p* < 0.0001, Fig 4). The rate of admissions within 4 months gradually increased from 30.0% (1995–1998) to 52.8% (2009–2013; *p* < 0.001). The rate after 8 months declined from 42.0% (1995–1998) to 13.0% (2009–2013; *p* < 0.001).

#### Age-wise comparison at time of injury

**Fig 5:**
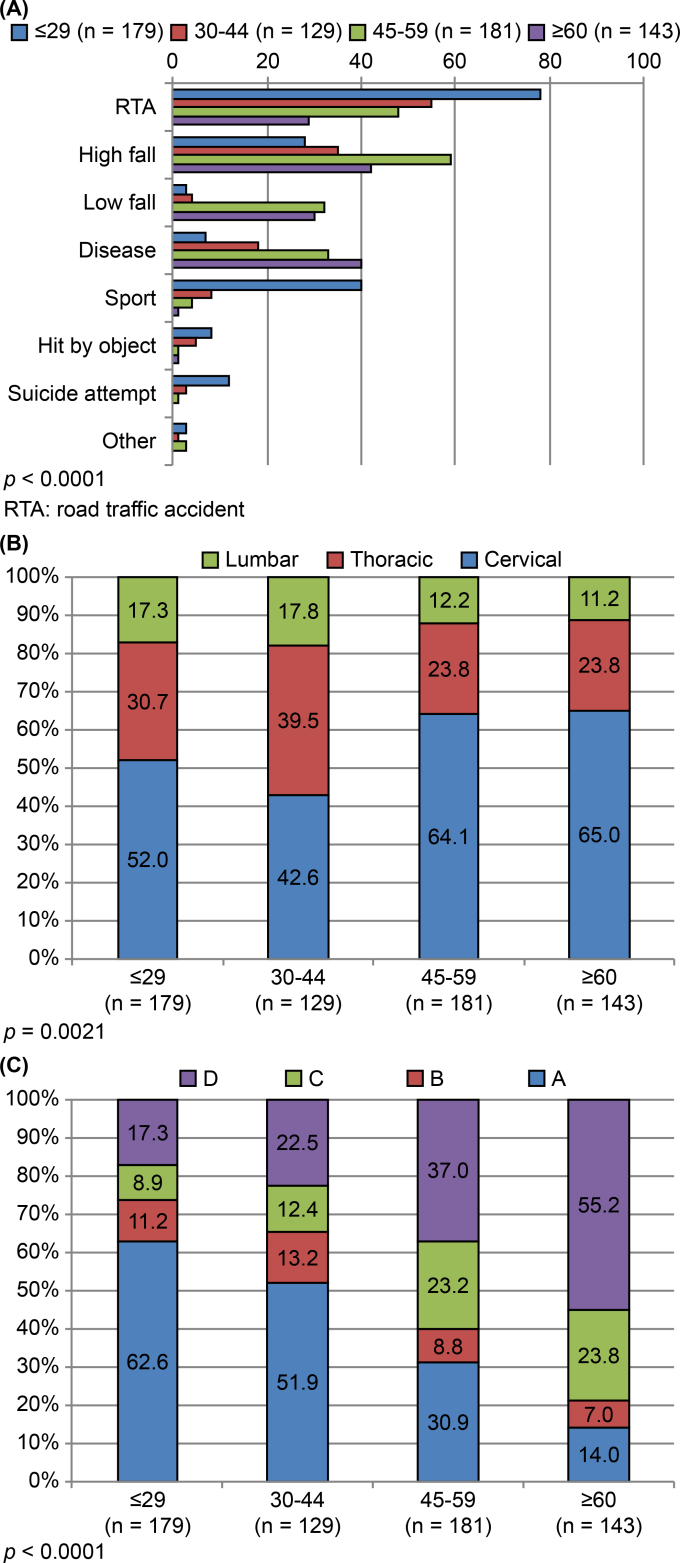
(A) Age-group distributions for various causes of spinal injury. (B) Distributions of spinal injury levels within patient age groups. (C) Distributions of AIS scores within patient age groups.

The average age (± SD) at injury for RTAs, high-distance falls, low-distance falls, diseases, sports, impact by objects, and suicide attempts were 38.2 ± 16.3, 47.6 ± 15.7, 57.6 ± 11.6, 54.5 ± 15.6, 26.5 ± 12.4, 33.2 ± 11.2, and 29.3 ± 10.5 years, respectively. S3 Table and Fig 5A–5C provide summaries of patient characteristics, stratified by age group at the time of injury. Patients ≤ 44 years old accounted for the majority of SCIs due to RTAs (63.3%, 133/210), sports (90.6%, 48/53), impact by objects (86.7%, 13/15), and suicide attempts (93.8%, 15/16) (Fig 5A). Sports (75.4%, 40/53) and suicide attempts (75.0%, 12/16) were the primary causes of SCIs in patients ≤ 29 years old. In patients ≥ 45 years old, SCIs were primarily caused by low-distance falls (62/69, 89.9% of all low-distance falls) and diseases (73/98, 74.5% of all diseases).

The frequency distributions for levels of injury differed significantly among age groups (*p* = 0.002, Fig 5B). Cervical injuries (resulting in tetraplegia) were relatively more frequent in patients ≥ 45 years old (58.5%, 209/357), whereas thoracic and lumbar injuries (paraplegia) occurred at higher rates in patients < 45 years old (58.2%, 160/275), and gradually decreased with advancing age (Fig 5B). In terms of AIS, the majority of AIS-A/B (motor complete injury) scores were present in patients ≤ 44 years old (67.9%, 216/318), and their proportion gradually declined as the age at injury advanced. Conversely, the rates of AIS-C/D (motor incomplete injury) scores gradually increased as the age at injury advanced. Patients ≥ 45 years old accounted for the majority of AIS-C/D scores (70.7%, 222/314), with AIS-D scores being predominant in patients ≥ 60 years old (55.2%, 79/143) (Fig 5C).

### Complete vs. incomplete injuries

Table 1 outlines patient characteristics, stratified by complete (AIS-A) and incomplete (AIS-B, -C, and -D) injuries. The frequency distributions by age at injury differed significantly for patients with complete and incomplete injuries (*p* < 0.0001). Complete injuries accounted for 70.2% (179/255) of SCIs in patients aged ≤ 44 years and 51.0% (130/255) of paraplegic injuries. However, incomplete injuries accounted for the majority of SCIs in patients ≥ 45 years old (65.8%, 248/377) and 32.6% of those sustained by patients ≥ 60 years old, while the majority were tetraplegic injuries (61.5%, 232/377). The frequency distributions according to cause of injury also differed significantly for complete and incomplete injuries (*p* < 0.0001). RTAs, high-distance falls, and impact by objects (i.e., high-energy trauma) were significantly more often implicated in complete (70.6%, 180/255) than in incomplete (55.4%, 209/377; *p* < 0.001) injuries; whereas low-distance falls and diseases (i.e., NTSCIs) corresponded less with complete (12.1%, 31/255) than with incomplete (36.1%, 136/377; *p*<0.001) injuries. Likewise, the frequency distributions by injury level differed significantly for complete and incomplete injuries (*p*<0.0001). Cervical injuries (tetraplegia) were significantly more common in patients with incomplete than complete injuries (61.5% vs. 49.0%; *p* < 0.01). Conversely, rates of thoracic and lumbar injuries (resulting in paraplegia) were significantly higher in patients with complete than in those with incomplete injuries (51.0% vs. 38.5%; *p* < 0.01). Finally, the frequency distributions for the period from injury to admission differed significantly for complete and incomplete injuries (*p* = 0.0004). Patients who were admitted within 4 months after injury displayed a significantly higher rate of incomplete than complete injury (43.8% vs. 32.2%; *p* < 0.01).

**Table 1:** Patient comparison by extent of spinal cord injury.

Patient variable	Extent of injury		*P*-value
	Complete	Incomplete	
	(n = 255)	(n = 377)	
Sex			0.759
Male	210 (82.4)	314 (83.3)	
Female	45 (17.6)	63 (16.7)	
Age at injury, years			<0.0001
≤ 29	112 (43.9)	67 (17.8)	
30–44	67 (26.3)	62 (16.4)	
45–59	56 (22.0)	125 (33.2)	
60–74	19 (7.5)	107 (28.4)	
≥ 75	1 (0.4)	16 (4.2)	
Cause of injury			<0.0001
RTA	106 (41.6)	104 (27.6)	
High fall	67 (26.3)	97 (25.7)	
Low fall	8 (3.1)	61 (16.2)	
Disease	23 (9.0)	75 (19.9)	
Sport	36 (14.1)	17 (4.5)	
Hit by object	7 (2.7)	8 (2.1)	
Suicidal attempt	6 (2.4)	10 (2.7)	
Other	2 (0.8)	5 (1.3)	
Level of injury			<0.0001
Cervical	125 (49.0	232 (61.5)	
Thoracic	113 (44.3)	70 (18.6)	
Lumbar	17 (6.7)	75 (19.9)	
Time from injury to admission, months			0.0004
< 4	82 (32.2)	165 (43.8)	
4–7	88 (34.5)	136 (36.1)	
≥ 8	85 (33.3)	76 (20.2)	

All data expressed as n (%)

Complete injury = AIS-A; Incomplete injury = AIS-B, -C, -D

RTA: road traffic accident; AIS: American Spinal Injury Association (ASIA) impairment scale

#### Traumatic vs non-traumatic SCIs

Table 2 outlines the patient characteristics stratified by traumatic and non-traumatic (disease-related) SCIs. In women, the rate of NTSCIs (33.7%) was significantly higher than traumatic SCIs (14.0%; *p* < 0.001). Patients who were ≥ 45 years old (especially those aged ≥ 60 years), accounted for the largest percentage of NTSCIs. Those aged ≤ 44 years sustained the majority of traumatic SCIs. Incomplete injuries were more often associated with NTSCIs (76.5%) than with traumatic SCIs (56.6%, *p* < 0.001), as were paraplegic injuries (NTSCIs: 78.6%, 77/98; traumatic SCIs: 37.1%, 198/534). In terms of the AIS status, the frequency distributions of AIS scores at admission differed significantly for traumatic and non-traumatic SCIs (*p* = 0.0023, Table 2). The rate of AIS-A was higher in traumatic SCIs than in NTSCIs (43.4% and 23.5%, respectively), whereas rates of AIS-B and -C were higher in patients with non-traumatic than traumatic SCIs (37.8% vs 25.8%, respectively). The rates of AIS-D were similar in both groups (NTSCIs, 38.8%; traumatic SCIs, 31.5%).

**Table 2:** Patient comparison by nature of spinal cord injury.

Patient variable	Nature of injury		*P*-value
	Non-traumatic-	Traumatic	
	(disease-related)		
	(n = 98)	(n = 534)	
Sex			<0.0001
Male	65 (66.3)	459 (86.0)	
Female	33 (33.7)	75 (14.0)	
Age at injury, years			<0.0001
≤ 29	7 (7.1)	172 (32.2)	
30–44	18 (18.4)	111 (20.8)	
45–59	33 (33.7)	148 (27.7)	
60–74	32 (32.7)	94 (17.6)	
≥ 75	8 (8.2)	9 (1.7)	
Level of injury			<0.0001
Cervical	21 (21.4)	336 (62.9)	
Thoracic	55 (56.1)	128 (24.0)	
Lumbar	22 (22.4)	70 (13.1)	
Admission AIS			0.0023
A	23 (23.5)	232 (43.4)	
B	13 (13.3)	50 (9.4)	
C	24 (24.5)	84 (15.7)	
D	38 (38.8)	168 (31.5)	
Time from injury to admission, months			0.2308
< 4	45 (45.9)	202 (37.8)	
4–7	28 (28.6)	196 (36.7)	
≥ 8	25 (25.5)	136 (25.5)	

All data expressed as n (%)

RTA: road traffic accident; AIS: American Spinal Injury Association (ASIA) impairment scale

## Discussion

### Patient characteristics

Our analysis included a total of 632 patients with SCIs who were admitted over a 19-year period. For reasons that are unclear, the number of admissions varied for each of the four established time-intervals (1995–1998, 1999–2003, 2004–2008, and 2009–2013). One likely influence was the great Hanshin-Awaji earthquake of January 1995. This quake hit the southern portion of Hyogo Prefecture, causing tremendous damage to the area and greatly affecting medical systems at our center [9]. Another potential influence was that, during the 2004–2008 and 2008–2013 periods, a new post-acute inpatient rehabilitation unit was introduced to the Japanese medical insurance [10]. This allows intensive rehabilitation of patients with SCIs within 180 days after injury. Several hospitals in Hyogo prefecture have since acquired such units, which may explain the trend for declining patient admissions at our center after 2004 (S2 Table).

### Sex and age

There was a nearly five-fold predominance of men in our study population, consistent with results of a prior Japanese study [2], and the age distribution was also similar. The number of elderly individuals (≥ 60 years old) consistently increased over the study period, corroborating recently generated data in the Tokushima prefecture [4].

### Cause of injury

Our analysis indicated that RTAs were the leading cause of SCIs during the study period (i.e., 1995–2013), which agreed with data from Canada and the United States [11,12]. High-distance falls were the second leading cause of SCIs, mostly sustained in younger age groups (Fig 5A). Nevertheless, such high-energy traumas tended to decline during the course of this study, as has previously been reported in other developed countries [13].

The frequencies of various diseases and low-distance falls, which were the third and fourth leading causes of SCIs in our study, respectively, however, have gradually increased during recent years, becoming the predominant cause of injury in the elderly (Figs 2 and 5A). Incomplete cervical lesions often resulted from low-distance falls (Table 1). This trend has also been reported by many developed countries worldwide, reflecting a distinct increase in the aging population of Japan [11,12,14–18].

Sports and suicide attempts were notable causes of SCIs in individuals aged ≤ 29 years, underscoring the need for preventative interventions in younger sectors of the population. Despite the launch of an injury-prevention campaign in Japan [3], sports-related injuries still accounted for approximately 8–9% of SCIs at our center during the study period, and thus remain an ongoing problem. Middle-aged and older adults (≥ 45 years) mostly had SCIs due to low-distance falls and disease (Fig 5A); hence, the prevention of low-distance falls is important in older adults. The marked expansion observed in the aging population of Japan, and the inherently narrow spinal canals in older individuals, and the high incidence of OPLL emphasize the urgency of this matter [6,19,20].

### Level and extent of injury (tetraplegia/paraplegia)

The rate of tetraplegia showed a slight decline (from 63% to 55%) during the study period; this trend was inconsistent with previous reports in Japan. Other sources have recorded a 75% rate of tetraplegia in SCIs [2], with > 90% of traumatic SCIs resulting in tetraplegia [4]. While these studies were confined to traumatic SCIs, both traumatic and NTSCIs were included in the present study. Thus, the declining trend could be attributed to the fact that nearly 80% of NTSCIs, which had recently increased, led to paraplegia (Table 2) and that many low-distance falls caused severe thoracic or lumbar burst fracture, resulting in paraplegia.

The rate of complete injury (AIS-A) has decreased in recent years, relative to earlier data (Fig 3). Approximately 70% of complete injuries could be attributed to high-energy traumatic events, such as RTAs and high-distances falls. Furthermore, the rate of paraplegia was significantly higher (Table 1), corroborating a previous epidemiological report in Japan [2]. Incomplete injuries, which were more prevalent in elderly people, were increasingly caused by low-distance falls or disease (Tables 1 and 2). In a recent Japanese study, more than 60% of SCIs during 2011 and 2012 were categorized as AIS-D, while AIS-A only accounted for 2.3% of cases [4]. The rate of AIS-D injuries at our center has increased, reaching 40% at latest recording (2009–2013). The remaining 60% (AIS-A, -B or -C) were associated with severe motor paralysis and impaired activities of daily living [7]. The reasons for this shift are uncertain. There may have been a selection bias, with severely paralyzed patients compelled to report to our specialized center. Certainly, the existing deadline for admission to a recovery-phase rehabilitation unit may be a contributing factor. Although patients must be admitted to such units within 60 days of injury [10], those with severe paralysis or complications requiring extended initial treatment may instead be referred to our specialist center. The fact that the rate of complete injuries in patients admitted within 4 months of onset was significantly lower than the rate of incomplete injuries supported this premise.

### Non-traumatic SCIs

Our analysis demonstrated that degenerative conditions, vascular diseases, and tumors accounted for the greater proportion of NTSCIs. Furthermore, NTSCIs were more likely to result in paraplegia. These results were in agreement with reports from countries such as the USA, Australia, and Denmark [21–23]. Additionally, there was a trend for patients with NTSCIs in the current study to increase in number and to be older than patients with traumatic SCIs, which also agreed with the findings of previous reports on NTSCIs [24]. Compared with accounts of traumatic SCIs, there are relatively few publications on NTSCIs in Japan [25]. Thus, further epidemiological investigation of NTSCIs is warranted to facilitate healthcare planning and delivery and formulation of preventative strategies.

## Conclusions

No previous study has reported the epidemiology of patients with SCIs in Japan. Given that the age demographics of Hyogo prefecture roughly mirror those of Japan overall [6,19], our findings could potentially provide insights into nationally developing trends. This report detailed the causes of traumatic SCIs and NTSCIs, enhancing our understanding of this condition through the longitudinal investigation of a large number of patients. Thus, this may prove useful in devising appropriate preventive strategies.

The chief limitation of the current study was the potential for selection bias implicit in a single-center analysis. Additionally, since not all patients with SCIs in the prefecture were referred to our Center, a regional estimate of SCI prevalence was not possible. However, we demonstrated that the patients’ age at the time of the SCI, as well as the injury cause, level, and extent, are interrelated. Thus, age-stratification may provide the best means of preventing/reducing the incidence of SCI in the Hyogo prefecture, and perhaps, throughout Japan.

## Supporting information

S1 Table (XLSX)

S2 Table (DOCX)

S3 Table (DOCX)
