# Supplementary material for: Age-specific characterization of spinal cord injuries over a 19-year period at a Japanese rehabilitation center
Source: PLoS One. 2018 Mar 29;13(3):e0195120. doi: 10.1371/journal.pone.0195120 (PMC5875854; doi:10.1371/journal.pone.0195120)
Supplement: S2 Table — (DOCX) [file pone.0195120.s002.docx]

S2 Table. Patient characteristics by year of injury

| **Patient variable** | **Case total** | **Year of injury** | | | | ***P*-value**  < 0.001 |
| --- | --- | --- | --- | --- | --- | --- |
|  |  | **1995−1998**  (n = 100) | **1999−2003**  (n = 253) | **2004−2008**  (n = 171) | **2009−2013**  (n = 108) |  |
| Sex  Male  Female | 524 (82.9)  108 (17.1) | 88 (88.0)  12 (12.0) | 208 (82.2)  45 (17.8) | 144 (84.2)  27 (15.8) | 84 (77.8)  24 (22.2) | 0.2481 |
| Level of injury  Cervical  Thoracic  Lumbar | 357 (56.5)  183 (29.0)  92 (14.6) | 63 (63.0)  28 (28.0)  9 (9.0) | 145 (57.3)  69 (27.3)  39 (15.4) | 90 (52.6)  57 (33.3)  24 (14.0) | 59 (54.6)  29 (26.9)  20 (18.5) | 0.3884 |

All data are expressed as n (%)
