# Supplementary material for: Age-specific characterization of spinal cord injuries over a 19-year period at a Japanese rehabilitation center
Source: PLoS One. 2018 Mar 29;13(3):e0195120. doi: 10.1371/journal.pone.0195120 (PMC5875854; doi:10.1371/journal.pone.0195120)
Supplement: S3 Table — (DOCX) [file pone.0195120.s003.docx]

S3 Table. Patient comparison by age at time of injury

| **Patient variable** | **Age at injury, years** | | | | ***P*-value** |
| --- | --- | --- | --- | --- | --- |
|  | **≤ 29**  (n = 179) | **30−44**  (n = 129) | **45−59**  (n = 181) | **≥ 60**  (n = 143) |  |
| Sex  Male  Female | 147 (82.1)  32 (17.9) | 108 (83.7)  21 (16.3) | 153 (84.5)  28 (15.5) | 116 (81.1)  27 (18.9) | 0.8501 |
| Time from injury to admission, months  < 4  4**−**7  ≥ 8 | 69 (38.5)  58 (32.4)  52 (29.1) | 45 (34.9)  50 (38.8)  34 (26.4) | 74 (40.9)  59 (32.6)  48 (26.5) | 59 (41.3)  57 (39.9)  27 (18.9) | 0.3755 |

All data are expressed as n (%)
